# Differences in factors associated with anemia in Haitian children from urban and rural areas

**DOI:** 10.1371/journal.pone.0247975

**Published:** 2021-04-06

**Authors:** Ana M. Palacios, Jeanne H. Freeland-Graves, Sherlie Jean-Louis Dulience, Jacques Raymond Delnatus, Lora L. Iannotti

**Affiliations:** 1 Department of Applied Health Science, School of Public Health, Indiana University, Bloomington, IN, United States of America; 2 Meds and Food for Kids, Haiti; 3 Institute for Public Health, George Warren Brown School of Social Work, Washington University in St. Louis, St. Louis, Missouri, United States of America; Helen Keller International, SIERRA LEONE

## Abstract

**Background:**

In Haiti, differences in the prevalence of anemia between urban and rural areas have been observed.

**Objective:**

To identify moderating factors that may help explain the difference in the prevalence of anemia in children from poor urban *vs*. rural areas of Haiti.

**Methods:**

This cross-sectional study used secondary data from urban and rural school-based trials that assessed the effectiveness of a nutrition intervention. The study was registered at ClinicalTrials.gov as NCT02747524. A total of 300 rural- and 981 urban- children between 2.5–13 years of age were included in this analysis. Effect modification in a binary logistic generalized linear mixed model was conducted using sample weights in SPSS^®^ version 26. Models were adjusted for age and income. School cluster was included as random effect.

**Results:**

In rural areas, stunting was more prevalent in children with anemia *vs*. no anemia, (16.6%, and 6.3%, P = 0.008), respectively. Also, rural children with anemia lived with fewer adults *vs*. rural children with no anemia, ($\bar{x}$ = 2.83±1.29, and 3.30±1.54, P = 0.005), respectively. In poor urban areas, helminth morbidities were more frequent in children with anemia *vs*. no anemia, (21.9% vs. 13.9, P = 0.011), respectively. In the combined sample, stunting, [AOR = 2.05; 95%CI (1.32–3.18)], age [AOR = 0.89; 95%CI (0.85–0.93)], and households with more adults [AOR = 0.77; 95%CI (0.67–0.87)] were associated with anemia. Effect modification by place of residence was observed in households with more adults (t = 3.83, P<0.001). No other nutritional, dietary, sanitation or morbidity factors or effect modifiers were observed.

**Conclusions:**

In this sample, factors associated with anemia differed in poor urban and rural children from Haiti including family structure and helminth morbidities. Stunting and lower age increased the odds of anemia in the combined sample. Family structure appears to have an important role in anemia, and further research understanding the influence of family structures in anemia is needed.

## Introduction

Haiti is the poorest country of the Americas and the Caribbean, and one of the countries with the highest income inequality in the world (Gini of 60.8) [1]. More than half of the population lives under the poverty line, and the economy is heavily dependent on foreign aid. In terms of health, Haiti has one of the highest prevalence rates of anemia (defined using the hemoglobin (Hb) cutoffs of the World Health Organization from 2011) [2]. In 2018, the prevalence was 66.3% for children under the age of 5 years (Hb <11.0 g/dL), and 49.0% for women ages 15 to 49 years (Hb <12.0 g/dL) [3, 4]. In addition, 21.9% of children were stunted (i.e. height-for-age z score <2) and 9.5%, wasting (i.e. weight-for-height <-2).

Young children and women are particularly vulnerable to nutritional deficiencies. The largely irreversible and negative long-lasting impacts of undernutrition and anemia during gestation and throughout the first years of life [5] provides compelling rationale for the focus on these specific subgroups. However, school-aged children living in poverty are still vulnerable, and nutritional deficiencies during childhood have been associated with poor academic and cognitive performance, lower work capacity, impaired learning and cognitive development [6–9].

A considerable body of literature worldwide reports that children residing in urban locations tend to have better nutritional and health status, as compared to those living in rural areas [10, 11]. A few studies from Haiti have documented higher levels of anemia in women and children in urban vs rural areas [12–14]. In previous analyses, we found that among children from urban, resource-poor communities, vitamin A supplementation and deworming were positively associated with increased hemoglobin concentrations [13]. Fever showed a negative association with hemoglobin, and stunting increased the odds of anemia by 1.48, 95% CI (1.05–2.08) in this population of Haitian children from urban residence [13]. At present, anemia in rural children from Haiti remains poorly characterized.

The discrepancy of anemia between urban and rural locations has been previously documented [12]. A study by Heidkamp *et al*. [12] observed a significantly larger proportion of anemia in Haitian young children from urban vs. rural areas; 65% vs. 56%, respectively [12]. To our knowledge, the differences in the prevalence of anemia in urban vs. rural children from Haiti have not been examined.

The objectives of this manuscript are to identify the differences in factors associated with anemia between poor children living in urban and rural areas (75.4% vs. 52.3%, respectively). and to identify how place of residence may moderate factors associated with anemia in this population.

## Methods

A secondary cross-sectional analysis of data was performed. The original studies tested the efficacy of a fortified peanut-based food product on child growth and anemia status in a population of 3- to 13-year-old children from poor communities in Cap-Haitien and poor rural areas (~120 km south) [15, 16]. Both studies were approved by the National Bioethics Committee of the Ministry of Health in Haiti and the Institutional Review Board of the Human Research Protection Office of Washington University in St. Louis, as one trial with a modification to include the rural site. The present research was approved by the University of Texas at Austin Institutional Review Board FWA # 00002030. Findings from the original trials have been published [14, 17] and the ClinicalTrials.gov registration number is NCT02747524.

### Study design and participants

**Fig 1:**
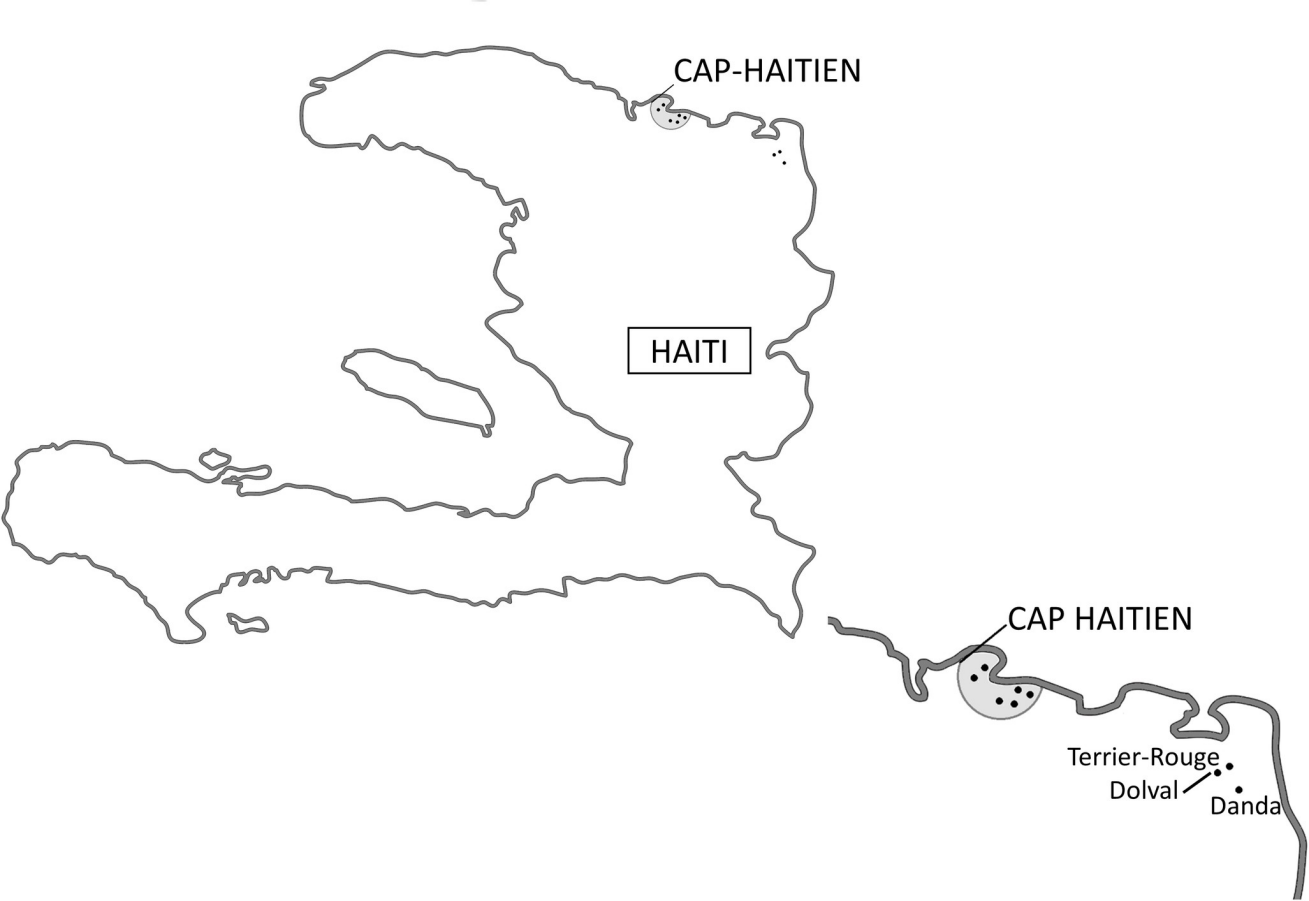
Map of Haiti that shows school locations in urban and rural areas. Modified from Worldatlas.com.

The urban study was conducted in Cap-Haitien with an estimated population of 500,000 inhabitants. The city is located in the Nord department by the Mapou River [18]. The schools were located in the following areas of the city: Petite-Anse, Cité Elie Lescot, and Cité Sainte-Philomène (Fig 1) which are poor, overcrowded areas. For this analysis, only baseline data collected in December 2012, was utilized. Details on school selection, eligibility and CONSORT diagram have been described elsewere [17]. Briefly, a total of six schools were included, and participants were invited to participate based on the following criteria: ages 2.5 to ≤13 years; no fever, congenital health condition, or peanut or soy allergy; not suffering from severe wasting (i.e. weight-for-height z-score <−3); registered at school in 2012–13, and having completed the baseline measurements and survey. A total of 1,186 children were assessed for eligibility in the six urban schools, 1,169 met eligibility criteria, and 981 had complete baseline measurements and survey. This trial was not registered in clinicaltrials.gov because at the time the trial was done, registration was not required by the funders.

The rural study was conducted in three schools from two poor rural communities (Dolval and Danda, Terrier-Rouge, Département Nord-Est), approximately 120 km from Cap-Haitien (Fig 1). Based on formative research, the communities and schools were considered representative of the Haitian rural livelihood for external validity. Details on school eligibility and CONSORT diagram have been described elsewere [14]. Data was collected in December 2014. Children from rural areas were then selected based on the same eligibility criteria as the urban trial, and school registration for the year 2014–15. A total of 442 children from rural schools were assessed for eligibility and 142 children did not meet age criteria, thus a total of 300 children attending the three rural schools were included in the present analysis.

For the present analysis, children from both trials between 2.5 to ≤13 years old were included, in order to have the same age ranges from both samples.

For both trials, written consent forms were provided to the parents and read aloud in case of illiteracy. The field staff collected signatures or crosses from the parents that agreed to participate in the study. Children were also asked to give verbal assent to participate in the trials.

### Measurements

A survey that included socioeconomic, demographic, water, sanitation, hygiene, child diet, and morbidity concerns was administered. Morbidity was determined with a 15-day recall for diarrhea (≥3 semi-solid or liquid stools in 24 hours) and fever. Monthly recalls were used for intestinal worm morbidities, cough, and ear infections; and 3 month recalls for malaria. Income and wealth questions included reception of money transfers from abroad, income, and an asset questionnaire [17]. Dietary information was collected through a 24-hour food frequency of intake using common foods identified in the formative research stage [19].

Drawing from formative research conducted in the sites, food lists were developed, and foods grouped into 17 categories. These included cassava bread, white wheat bread; cereals, (rice, maize, wheat, millet, sorghum, spaghetti, and other pastas); roots and tubers (cassava, potatoes, yams, cooking bananas, other); beans and other legumes (beans, peas, groundnuts, grams, other); eggs; milk (cow, goat); yogurt; cheese and other dairy products; poultry (chicken, duck, guinea fowl, other); meat (beef, goat, pork, lamb, other); fish and shellfish (large and small fish); fruits; vegetables; oils, butter, and other fats; crackers; and cookies. Dietary diversity was calculated by first generating dichotomous variables for whether or not the child had consumed each food group listed above, and then by adding the food types consumed in the previous 24 hours [20]. The score ranged from 0 to 17, and higher numbers represented more diverse diets. The “fresh fruits and vegetables” variable was determined by adding consumption of the “fruits” and “vegetables” food groups. The “animal source foods” variable was created by adding “meat, fish and shellfish, yogurt, cheese and other dairy products, eggs and poultry.” The variable “owns livestock” consisted of affirmative answers to questions concerning owning poultry, cows, horses, mules, and goats/pigs.

Hemoglobin was collected from capillary blood by trained staff and analyzed in the field, using a previously calibrated portable spectrophotometer (Hemocue 201a, Brea, CA), following the manufacturer’s instructions [21]. Standing height was measured with a stadiometer (ShorrBoard, Weigh and Measure, LLC, Olney, MD) to the nearest 1 mm. Weights were measured to the nearest 0.1 kg, with a digital weight scale (Seca Model 874, Chico, CA) following standard protocols [22]. Anemia was defined using the World Health Organization Hb thresholds for each age group [2]: children 6 months to <5 years: Hb <11.0 g/dL); children 5 to <12 years Hb <11.5 g/dL); and children 12–14 years Hb<12.0 g/dL.

Anthropometric z-scores were calculated based on the 2006 World Health Organization’s Child Growth Standards for children <5 years of age, and Child Growth References for older children [23, 24].

Child morbidity was evaluated via caregiver monthly recalls for the following morbidities: malaria, fever, respiratory symptoms (cough and short, rapid breathing) and worm infection. Recall for diarrhea (three or more liquid or semisolid stools in 24-hours), was assessed in a two-week period.

### Statistical analysis

Data collected was entered into a database using SPSS^®^ statistical software package version 26.0 (IBM SPSS Inc., Chicago, IL, USA) for this analysis [14, 17].

A literature review was performed to identify sociodemographic, dietary and maternal/child variables that showed a relationship with anemia and place of residence (dependent variables). Variables available from the survey and that were previously identified in the literature review were included in a univariate analysis with simple binary logistic regressions to identify factors associated with anemia in urban and rural areas. Factors included in this first step are listed in Table 1.

**Table 1:** Sociodemographic and morbidity characteristics of urban and rural Haitian school-aged children^1^^,^ ^2^.

	Urban			Rural		
	Anemia			Anemia		
	No (n = 241)	Yes (n = 740)	*P*	No (n = 143)	Yes (n = 157)	*P*
***Child Characteristics***						
Anemia, %	24.6	75.4	0.001	47.7	52.3	
Hb, g/dL	12.16 ± 0.64	10.05 ± 1.15		12.28 ± 0.77	10.15 ± 0.93	
Age, yr	8.35 ± 2.37	8.20 ± 2.50	0.411	8.01 ± 2.71	7.22 ± 2.89	**0.017**
Female sex, %	51.9	51.6	0.947	45.1	50.3	0.365
***Sociodemographic***						
Maternal education (≥ secondary), %	36.9	36.4	0.899	33.8	32.9	0.870
Income (>501 Gourdes)^3^, %	34.2	33.6	0.883	46.1	39.6	0.261
Living in household, N						
Children	3.29 ± 1.54	3.36 ±1.64	0.604	4.04 ± 1.70	3.92 ± 1.64	0.535
Adults	2.90 ± 1.59	3.10 ± 2.06	0.204	3.30 ± 1.54	2.83 ± 1.29	**0.005**
Livestock ownership, %	19.8	17.3	0.411	75.4	72.0	0.509
***Water and Sanitation***						
Tap water at home, %	25.8	25.6	0.945	3.5	3.2	0.872
Open defecation or other	18.3	18.2	0.996	32.9	33.8	0.870
Treats drinking water, %	69.6	69.3	0.942	82.0	83.4	0.755
Number of people using same latrine	8.77 ± 4.12	9.40 ± 4.50	0.080	8.30 ± 2.60	8.30 ± 3.38	0.995
***Nutritional and Health Status***						
Stunted, %	11.1	15.0	0.137	6.3	16.6	**0.008**
Underweight, %	11.3	15.5	0.172	6.8	15.1	0.055
Fever (past 2 weeks), %	19.2	25.1	0.067	6.3	4.5	0.481
Malaria (past 3 months), %	7.5	10.1	0.219	0.0	0.6	1.00
Diarrhea (past 2 weeks), %	15.4	11.0	0.070	3.5	2.5	0.624
Intestinal worm morbidities (past 1 month), %	13.9	21.9	**0.011**	1.4	0.7	0.524
Deworming treatment (past 6 months), %	74.5	78.0	0.286	65.0	64.7	0.963
***Dietary***						
Dietary diversity score	4.67 ± 2.12	4.65 ± 2.28	0.937	4.42 ± 1.59	4.37 ± 1.71	0.790
Any animal source food, %	37.5	42.1	0.235	27.9	23.9	0.429
Cereals, %	86.1	84.8	0.641	90.8	89.7	0.750
Fresh fruits and vegetables, %	52.8	51.1	0.673	51.8	57.1	0.366
Vitamins (past 6 months), %	28.6	25.2	0.293	13.8	20.7	0.220

^1^ Values are means (SDs) unless otherwise indicated.

^2^ Groups significantly different by unadjusted univariate binary logistic regressions.

^3^ 501 Haitian Gourdes was equivalent to ~10 USD at the time of data collection (December, 2012 and 2014).

To examine the relationship between factors associated with anemia by place of residence effect modification was applied in the combined sample using interaction terms in a logistic generalized linear mixed model with binary outcome (GLMM) to identify whether place of residence moderated variables associated with anemia (dependent variable). Variables included in this model were those identified as significantly associated with anemia in either rural or urban areas in the bivariate analysis shown in Table 1 (helminth infection morbidities for urban areas, stunting, and number of adults in the household for rural locations). However, in the final model, only the significant interactions were retained. The equation was adjusted for potential confounders, including age and income as fixed effects (Table 2). To avoid an inflation of type I error rates in the results from this study, the school clusters were included in the GLMM as random effects [25]. The urban and rural samples were weighted based on the proportion of inhabitants under 15 years of age (35.9%) estimated to live in rural (43.81%) and urban (56.19%) regions in Haiti, using data from the World Bank (total population = 11,085,919).

**Table 2:** Effect modification with logistic generalized linear mixed model in anemia^1^.

Variable	AOR (95% CI)	P
Age	**0.89 (0.85–0.93)**	**<0.001**
Monthly income^2^	0.76 (0.58–1.002)	0.052
Place of residence	0.71 (0.23–2.13)	0.536
Helminth morbidities	1.54 (0.94–2.52)	0.084
Adults in household	**0.77 (0.67–0.87)**	**<0.001**
Adults in household*	1.07 (0.96–1.19)	0.233
Stunting	**2.05 (1.32–3.18)**	**0.001**
Adults in household * place of residence	**1.40 (1.18–1.66)**	**<0.001**

^1^ Adjusted odds ratios (AOR) are provided for the combined sample, all variables unless otherwise indicated

(*) are showing the effect when rural areas are coded as “1” and urban areas coded as “0”.

^2^ Households with 501 or more Haitian Gourdes (equivalent to ~$10 at the time of data collection, December 2012 and 2014).

## Results

A total of six schools from urban areas with n = 981 eligible students, and three schools from rural areas with n = 300 eligible participants were included in this analysis, n = 1,281). The intra-cluster correlation coefficient between school clusters was of 0.08. Characteristics of urban and rural children are listed in Table 1. Anemia was present in more than one-half of Haitian children in both urban and rural areas. The prevalence was significantly higher in urban children compared to rural, 75% and 52%, respectively, P<0.001. The unadjusted OR for anemia for urban children was 2.80; 95% CI (2.14–3.66) and for rural children was 0.36; 95% CI (0.27–0.47).

### Anemia vs. no-anemia

In urban areas, intestinal helminth morbidities were significantly higher in children with anemia, 21.9%, relative to children with no anemia, 13.9%, P = 0.011. For rural children, age, and number of adults in the household were both lower in children with anemia (7.22 years ± 2.89, vs. 8.01 years ± 2.71, P = 0.017), and (2.83 adults ± 1.29, and 3.30 adults ± 1.54, P = 0.005). Stunting was more frequent in children with anemia, 16.6% vs. no anemia 6.3%, P = 0.008. No other comparisons between children with anemia, relative to children with no anemia yielded significant results.

### Urban vs. rural

Comparisons between urban and rural children show that urban children were about 6 months older than those living in rural areas, P<0.001. The 33.8% of urban households reported an income of ≥501 Gourdes (equivalent to approximately 10 USD). Significantly more rural households reported incomes above 501 Gourde, 42.7%, P = 0.005. Greater income was associated with livestock ownership [OR 1.37; 95%CI (1.01–1.84), P = 0.041], even after adjusting by residence and school cluster.

The ratio of people per room was significantly greater in urban vs. rural areas (median of 3.0 for urban and 2.0 for rural, P<0.001). The number of rooms was lower in urban households by a magnitude of one. As expected, about one in seven urban households owned poultry, as compared to one in two rural households, P<0.001.

Urban homes had eight times greater access to tap water than did rural households. However, fewer urban participants reported always treating drinking water through chlorine/aquatabs or boiling water, as compared to rural: 69.3% vs. 82.8%, P<0.001, respectively. The number of people using the same latrine was significantly higher in urban vs. rural households, P<0.001.

In urban areas, malaria was observed in the 10.1% of children with anemia, and in the 7.5% of children with no anemia. The reported prevalence of malaria was below 1% in rural locations.

A significantly larger proportion of urban children received deworming treatment (77.2%) in the past 6 months, as compared with those from rural areas (64.9%), P<0.001.

Almost twice as many urban children reported eating foods from animal sources (40.1%), compared to 25.8% from rural areas, P<0.001. Supplementation with iron or vitamin A in the previous 6 months was more frequent in urban children, compared to rural, 26.0% vs. 17.2%, P = 0.011, respectively.

### Effect modification of place of residence in factors associated with anemia

Table 2 shows the generalized linear mixed model using the combined sample for urban and rural place of residence and anemia. The adjusted odds ratios shown are for children from rural areas. Effect modification by place of residence in anemia was significant for households with more adults (t = 3.83, P<0.001).

Stunting increased the odds of anemia in children from Haiti; however, interaction terms of these variables with place of residence were not significant.

For every additional year of age, the odds of anemia were reduced an 11% in the present sample.

## Discussion

The main purpose of this analysis was to identify the differences in factors associated with anemia between poor children living in urban and rural areas of Haiti, and to understand how place of residence may moderate these factors. Findings from this analysis show that effect modification by place of residence in anemia was observed for households with more adults. No other moderators were identified in this sample.

The proportion of anemia for urban children (75.4%) appears to be the highest reported for any Latin American and the Caribbean country or program in recent times [26]. Similar findings were described by Heidkamp *et al*. 2013 using data from a national representative sample from the Demographic Health Survey in 2005/6, where the prevalence of anemia in children under age 5 was significantly higher in urban areas, as compared with rural areas, (65.1 vs. 55.7%, P = 0.004), this trend was also observed for women of reproductive age (54.4 vs. 43.1%, P< 0.001) [12].

Several studies have suggested that household composition may have an important influence in child health, nutritional status and cognitive development [27, 28]. In Latin America and the Caribbean, complex household structures such as extended families are common [29]. In this analysis, the 49% and 53% of families from urban and rural areas, respectively had three or more adults living together. Previous studies in Uganda [30] and in rural Guatemala [31] reported that children living with larger numbers of adults had a lower probability to suffer anemia. Moreover, the study in Guatemala reported that larger households with more adults owned more assets, and mothers of young children living in these households received more years of education. Thus, it is plausible that additional adults, particularly in rural areas, may result in additional time, increased income, household space and resources that could be spent in the family wellbeing, especially in childhood health and care [32, 33]. Unfortunately, the original studies were not designed *a priori* to examine this question, thus further research of household composition and the influence in child anemia, and overall health is impending.

In some countries from the Western hemisphere, the urban poor can have equal or worse health outcomes than the rural poor [34]. To explain the urban-rural gap in anemia, this study hypothesized that increased infectious morbidity could influence anemia in urban vs. rural locations [35, 36]. In Haiti, urban areas are more populated and crowded compared to rural areas. This study found a significantly higher crowding index in urban areas. Furthermore, the schools included in our study were in poor communities of Cap-Haitien. Inadequate and overcrowded housing in highly-populated urban areas can favor the spread of parasitic infections [37, 38], tuberculosis [39], diarrheal diseases [40], respiratory infections [41] and other infectious diseases and fever [41], resulting in increased morbidity in overcrowded areas. For instance intestinal helminth morbidities were much more prevalent in urban children, as compared with rural children in the present analysis. However this variable does not appear to moderate the effect between place of residence and anemia.

Intestinal parasites are a primary cause of diarrhea, dysentery (blood associated with diarrhea) and intestinal malabsorption [42]. Other studies have reported high prevalences of helminth infections in Haiti. In children from the Nord department of Haiti, Champetier de Ribes reported a prevalence of helminth infection of 45.2%, [43], with a 28% prevalence of co-infection with two or more helminths [43]. These intestinal multi-parasitic infections elevate the probability of anemia even more so than single infections via hemorrage and chronic inflammation [44] and urban children may be more susceptible than children from rural areas due to the population density in urban areas [45].

This investigation also observed that children residing in urban areas reported fever, malaria, and diarrhea much more frequently than those from rural areas. These results are similar to the latest Haiti survey that documented a slightly higher morbidity rates in urban vs. rural children under 5 years: fever, 32.8% vs. 30.8%; and diarrhea, 22.6% vs. 20.5% [3]. Increased infectious burden is thought to promote anemia via chronic inflammation mediated by hepcidin activation, an acute phase protein that inhibits intestinal absorption of iron and reduces its release into the circulation [46, 47].

Another hypothesis proposed by Heidkamp *et*. *al*. [12] was that people from rural areas from Haiti would have greater access to seasonal micronutrient-rich fruits and vegetables at low- or no-cost, vs. those from urban locations. In contrast, urban populations may have had more access to energy-dense cereal staples [12]. Yet, the present research observed the opposite, with rural children having greater consumption of cereals. No differences in the intake of fresh fruits or vegetables were observed, based on area of residence (Table 1). This analysis also explored whether children residing in rural areas would have a greater intake of animal source foods. Animal source foods are rich sources of essential and bioavailable micronutrients that are difficult or impossible to obtain via plant sources. Potential problem nutrients might be vitamin A, vitamin B12, riboflavin, iron or zinc [48, 49]. In rural areas, the rearing of animals is an ubiquitous practice [50], and studies have linked livestock ownership with increased intake of meat and dairy [51] and better nutritional status in children [52]. As expected, a larger proportion of rural households owned livestock; however, intake of meat, dairy and eggs was more frequent in children from urban residence. Findings from this study are similar to a study in Ghana, where livestock ownership was not associated with increased intake of animal source foods [53].

On the contrary, livestock ownership has been associated with increased household incomes [51], as was observed in this research. Engle [54] suggested that increased income by itself may lower anemia either by improving dietary quality and/or by possibly increasing expenditures in other wellbeing activities [54]. It is plausible that resources in rural households may be directed towards improvements in water treatment, health and disease prevention, vector management, or other aspects that could ameliorate the anemia burden in rural children.

In this investigation, stunting was associated with anemia. Our findings are similar to those of Iannotti *et*. *al*., [13] who observed an association between stunting and anemia in a longitudinal analysis in urban children from Haiti.

Stunting is a functional indicator of chronic micronutrient deficiencies [55] and, like anemia, stunting has been associated with chronic inflammation [56]. Micronutrient deficiencies including iron, vitamin A, folate and vitamin B12, among others are known causes of anemia [36, 57]. The co-occurrence of stunting and anemia is common in low- and middle-income countries [58–60], and both have been associated with improper nutrition, lower education, poverty, lack of access to potable water, improper sanitation, and poor nutrition knowledge [61]. Regardless, a recent multi-country analysis observed that, although anemia and stunting may share some basic factors, both conditions may be more independent than commonly assumed. This suggests that interventions developed to address either condition, in isolation of the other, may fail to improve the other [62].

Inherited hemoglobin disorders are known contributors of anemia. A study implemented in a large hospital of Port-Au-Prince screened more than 2,258 newborns for Hb types F, A, S, and C in newborns and reported a prevalence of HbSS and HbSC of 1 in 173 Haitian newborns [63]. Furthermore, other studies examining the genetic structure of populations from Haiti observed that Haitians exhibited minimal contributions from non-African populations in their gene pool. This suggests that Haitians may reflect similar prevalences of hemoglobinopathies as do populations from Sub-Saharan Africa, which are highly prevalent [64]. Finally, a report from the Ouest department in Haiti observed a prevalence of approximately 20% of glucose 6 phosphate dehydrogenase deficiency in a population of school-aged children further suggesting that hemoglobinopathies may be prevalent in this population [65]. A limitation of this research is that inherited hemoglobin disorders were not contemplated in this sample.

Another limitation is that the urban and rural trials occurred in different years. Yet there does not appear to be any major environmental, socio-economic, or other significant events occurring in the two years that may have driven differences in anemia. Another limitation is that this study examined secondary data that was not selected randomly from a national sample. However, both urban and rural schools were selected after extensive formative research to ensure both communities and schools could be considered representative of the Haitian poor urban and rural livelihood for external validity. Furthermore, the lower numbers of children enrolled in urban compared to rural could have affected the observed differences, however weights were used to address this issue. Dietary and infectious information were reported by the primary caregivers, which could result in reporting biases. Also, intestinal worm morbidities were not confirmed by fecal sample analysis, such that intensity of infections could not be determined. Both could be potential sources of reporting bias, as well as an underestimation of the intestinal parasite morbidity burden. Also, this research did not collect nutritional or inflammatory biomarkers associated with anemia which could have provided insight into anemia etiologies as well.

In conclusion, this research suggests that non-nutritional factors may be central to the etiology of anemia in school-aged children from Haiti. To our knowledge, this is the first study that aims to explain the existing urban-rural differences in anemia rates among school-aged children from Haiti. Furthermore, this is the second study in Latin America and the Caribbean that associates households with more adults as a protective factor for anemia in rural areas, by offering stronger social support, time and additional resources for childcare and family wellbeing [32]. This information can facilitate the development and implementation of cost-effective programs and interventions targeted to improve the nutritional and health status of Haitian children based on their place of residence. As families appear to play an important role in the health status of children, future programs aiming to improve child’s health, nutrition and anemia in the region should consider using a more inclusive approach with additional family members (or even the whole family), in the delivery of the intervention. Furthermore, to reach additional family members, adding parenting and responsive caregiving components may be plausible in low resource settings without considerably increasing programmatic costs by utilizing existing platforms of community outreach (e.g., schools, community centers, churches). Still, further research should seek to understand family structures and household composition, and their relationship with child’s health and wellbeing in both urban and rural contexts.

## Supporting information

S1 Dataset Effect modification. (SAV)

S1 File Output effect modification. (HTM)

S2 File Study profile. (DOCX)
