# Supplementary material for: Differences in factors associated with anemia in Haitian children from urban and rural areas
Source: PLoS One. 2021 Apr 6;16(4):e0247975. doi: 10.1371/journal.pone.0247975 (PMC8023464; doi:10.1371/journal.pone.0247975)
Supplement: S1 File — (HTM) [file pone.0247975.s002.htm]

xml version="1.0" encoding="UTF-8"?


Output\_effect\_modification


|  |  |  |
| --- | --- | --- |
| IBM SPSS Web Report - Output\_effect\_modification.spv     ---   Contents  Previous  Next  Help |  | Controls disabled by the system     --- |

- Log

  - Log
- Table 2. Generalized Linear Mixed Models

  - Warnings
  - Case Processing Summary
  - Model Summary
  - Data Structure
  - Classification
    Overall Percent Correct = 73.3%
  - Fixed Effects
  - Fixed Effects
  - Fixed Coefficients
  - Fixed Coefficients
  - Random Effect Covariances

    - Random Effect Block 1
  - Covariance Parameters

    - Covariance Parameters Summary
    - Residual Effect
    - Random Effect
  - Estimated Marginal Means for Top Significant Fixed...

    - Anemia: <11 <5 yrs; <11.5 11.5 yrs; <1...
- Log

  - Log
- Table 2. urban\_rural\_Generalized Linear Mixed Mode...

  - Warnings
  - Case Processing Summary
  - Model Summary
  - Data Structure
  - Classification
    Overall Percent Correct = 73.3%
  - Fixed Effects
  - Fixed Effects
  - Fixed Coefficients
  - Fixed Coefficients
  - Random Effect Covariances

    - Random Effect Block 1
  - Covariance Parameters

    - Covariance Parameters Summary
    - Residual Effect
    - Random Effect
  - Estimated Marginal Means for Top Significant Fixed...

    - Anemia: <11 <5 yrs; <11.5 11.5 yrs; <1...
- Log

  - Log
- Including all intraction terms Generalized Linear ...

  - Warnings
  - Case Processing Summary
  - Model Summary
  - Data Structure
  - Classification
    Overall Percent Correct = 73.2%
  - Fixed Effects
  - Fixed Effects
  - Fixed Coefficients
  - Fixed Coefficients
  - Random Effect Covariances

    - Random Effect Block 1
  - Covariance Parameters

    - Covariance Parameters Summary
    - Residual Effect
    - Random Effect
  - Estimated Marginal Means for Top Significant Fixed...

    - Anemia: <11 <5 yrs; <11.5 11.5 yrs; <1...
- Log

  - Log
- Frequencies

  - Statistics
  - Respondent Occupation
- Log

  - Log
- Frequencies

  - Statistics
  - Spouse Occupation
- Log

  - Log
- Frequencies

  - Statistics
  - Respondent Occupation
- Log

  - Log
- Frequencies

  - Statistics
  - Spouse Occupation

- Delete

Log  
Log - Log - November 29, 2020

GENLINMIXED  
  /DATA\_STRUCTURE SUBJECTS=lekol1  
  /FIELDS TARGET=anemia TRIALS=NONE  ANALYSIS\_WEIGHT=scale\_weight OFFSET=NONE  
  /TARGET\_OPTIONS REFERENCE=0 DISTRIBUTION=BINOMIAL LINK=LOGIT  
  /FIXED  EFFECTS= age recode\_incnew rural\_urban recodeworm adultsliving recodestunted adultsliving\*rural\_urban  
  USE\_INTERCEPT=TRUE  
  /RANDOM USE\_INTERCEPT=TRUE SUBJECTS=lekol1 COVARIANCE\_TYPE=VARIANCE\_COMPONENTS  
  /BUILD\_OPTIONS TARGET\_CATEGORY\_ORDER=ASCENDING INPUTS\_CATEGORY\_ORDER=ASCENDING MAX\_ITERATIONS=100  
  CONFIDENCE\_LEVEL=95 DF\_METHOD=RESIDUAL COVB=MODEL PCONVERGE=0.000001(ABSOLUTE) SCORING=0 SINGULAR=0.000000000001.

Generalized Linear Mixed Models

WarningsWarnings, table, 0 levels of column headers and 0 levels of row headers, table with 1 columns and 4 rows

|  |
| --- |
| glmm: One or more records are not used in the analysis because they have one or more fields with invalid or missing values. |
| glmm: Valid values for events (target) and trials variables are non-negative and positive integers respectively, and the number of trials cannot be less than the number of events. |
| glmm: Analysis weight data values must be positive. |
|  |

Generalized Linear Mixed Models

Case Processing SummaryCase Processing Summary, table, 1 levels of column headers and 1 levels of row headers, table with 3 columns and 5 rows

|  |  |  |
| --- | --- | --- |
|  | N | Percent |
| Included | 1150 | 89.8% |
| Excluded | 131 | 10.2% |
| Total | 1281 | 100.0% |
|  |  |  |

Generalized Linear Mixed Models

Model SummaryModel Summary, table, 0 levels of column headers and 2 levels of row headers, table with 3 columns and 7 rows

|  |  |  |
| --- | --- | --- |
| Target | | Anemia: <11 <5 yrs; <11.5 11.5 yrs; <12 14 yrs |
| Probability Distribution | | Binomial |
| Link Function | | Logit |
| Information Criterion | Akaike Corrected | 5381.946 |
|  | Bayesian | 5386.983 |
|  |  |  |
| --- | --- | --- |
| Information criteria are based on the -2 log likelihood (5379.942) and are used to compare models. Models with smaller information criterion values fit better. | | |
|  |  |  |

Generalized Linear Mixed Models

Data StructureaData Structure, table, 2 levels of column headers and 1 levels of row headers, table with 3 columns and 16 rows

|  |  |  |
| --- | --- | --- |
|  | Subjects | Target |
|  | lekol1 | Anemia: <11 <5 yrs; <11.5 11.5 yrs; <12 14 yrs |
| Data for First Subject | 1 | Non-anemic |
|  | 1 | Anemic |
|  | 1 | Non-anemic |
|  | 1 | Anemic |
|  | 1 | Anemic |
|  | 1 | Non-anemic |
|  | 1 | Non-anemic |
|  | 1 | Non-anemic |
|  | 1 | Non-anemic |
|  | 1 | Anemic |
| Total Number of Levels | 9 |  |
|  |  |  |
| --- | --- | --- |
| Only the first 10 records are displayed.a | | |
| a. Target: Anemia: <11 <5 yrs; <11.5 11.5 yrs; <12 14 yrs | | |
|  |  |  |

Generalized Linear Mixed Models

Generalized Linear Mixed Models

Fixed EffectsaFixed Effects, table, 1 levels of column headers and 1 levels of row headers, table with 5 columns and 12 rows

|  |  |  |  |  |
| --- | --- | --- | --- | --- |
| Source | F | df1 | df2 | Sig. |
| Corrected Model | 7.490 | 7 | 1142 | .000 |
| age | 20.077 | 1 | 1142 | .000 |
| recode\_incnew | 3.780 | 1 | 1142 | .052 |
| rural\_urban | .384 | 1 | 1142 | .536 |
| recodeworm | 2.987 | 1 | 1142 | .084 |
| adultsliving | 5.381 | 1 | 1142 | .021 |
| recodestunted | 10.292 | 1 | 1142 | .001 |
| adultsliving \* rural\_urban | 14.692 | 1 | 1142 | .000 |
|  |  |  |  |  |
| --- | --- | --- | --- | --- |
| Probability distribution: Binomial Link function: Logita | | | | |
| a. Target: Anemia: <11 <5 yrs; <11.5 11.5 yrs; <12 14 yrs | | | | |
|  |  |  |  |  |

Generalized Linear Mixed Models

Generalized Linear Mixed Models

Fixed CoefficientsaFixed Coefficients, table, 2 levels of column headers and 1 levels of row headers, table with 10 columns and 19 rows

|  |  |  |  |  |  |  |  |  |  |
| --- | --- | --- | --- | --- | --- | --- | --- | --- | --- |
| Model Term | Coefficient | Std. Error | t | Sig. | 95% Confidence Interval | | Exp(Coefficient) | 95% Confidence Interval for Exp(Coefficient) | |
|  |  |  |  |  | Lower | Upper |  | Lower | Upper |
| Intercept | 2.195 | .5072 | 4.328 | .000 | 1.200 | 3.191 | 8.984 | 3.321 | 24.306 |
| age | -.117 | .0262 | -4.481 | .000 | -.169 | -.066 | .889 | .845 | .936 |
| recode\_incnew=0 | -.269 | .1383 | -1.944 | .052 | -.540 | .002 | .764 | .583 | 1.002 |
| recode\_incnew=1 | 0b | . | . | . | . | . | . | . | . |
| rural\_urban=0 | -.349 | .5627 | -.619 | .536 | -1.453 | .755 | .706 | .234 | 2.129 |
| rural\_urban=1 | 0b | . | . | . | . | . | . | . | . |
| recodeworm=0 | .433 | .2508 | 1.728 | .084 | -.059 | .925 | 1.542 | .943 | 2.523 |
| recodeworm=1 | 0b | . | . | . | . | . | . | . | . |
| adultsliving | -.268 | .0672 | -3.984 | .000 | -.400 | -.136 | .765 | .671 | .873 |
| recodestunted=0 | .719 | .2240 | 3.208 | .001 | .279 | 1.158 | 2.052 | 1.322 | 3.184 |
| recodestunted=1 | 0b | . | . | . | . | . | . | . | . |
| adultsliving\*[rural\_urban=0] | .334 | .0871 | 3.833 | .000 | .163 | .505 | 1.396 | 1.177 | 1.656 |
| adultsliving\*[rural\_urban=1] | 0b | . | . | . | . | . | . | . | . |
|  |  |  |  |  |  |  |  |  |  |
| --- | --- | --- | --- | --- | --- | --- | --- | --- | --- |
| Probability distribution: Binomial Link function: Logita | | | | | | | | | |
| a. Target: Anemia: <11 <5 yrs; <11.5 11.5 yrs; <12 14 yrs | | | | | | | | | |
| b. This coefficient is set to zero because it is redundant. | | | | | | | | | |
|  |  |  |  |  |  |  |  |  |  |

Generalized Linear Mixed Models

Random Effect Covariances  
Random Effect Covariances - Random Effect Block 1 - November 29, 2020

Random Effect Block 1Random Effect Block 1, table, 1 levels of column headers and 1 levels of row headers, table with 2 columns and 4 rows

|  |  |
| --- | --- |
| Random Effect Block | Intercept |
| Intercept | .437 |
|  |  |
| --- | --- |
| Covariance Structure: Variance components Subject Specification: lekol1 | |
|  |  |

Covariance Parameters

Covariance Parameters SummaryCovariance Parameters Summary, table, 0 levels of column headers and 2 levels of row headers, table with 3 columns and 8 rows

|  |  |  |
| --- | --- | --- |
| Covariance Parameters | Residual Effect | 0 |
|  | Random Effects | 1 |
| Design Matrix Columns | Fixed Effects | 13 |
|  | Random Effects | 1a |
| Common Subjects | | 9 |
|  |  |  |
| --- | --- | --- |
| Common subjects are based on the subject specifications for the residual and random effects and are used to chunk the data for better performance. | | |
| a. This is the number of columns per common subject. | | |
|  |  |  |

Covariance Parameters  
Covariance Parameters - Residual Effect - November 29, 2020

Residual EffectResidual Effect, table, 2 levels of column headers and 1 levels of row headers, table with 7 columns and 5 rows

|  |  |  |  |  |  |  |
| --- | --- | --- | --- | --- | --- | --- |
| Residual Effect | Estimate | Std. Error | Z | Sig. | 95% Confidence Interval | |
|  |  |  |  |  | Lower | Upper |
| Variance | 1.000 | . | . | . | . | . |
|  |  |  |  |  |  |  |
| --- | --- | --- | --- | --- | --- | --- |
| Covariance Structure: Scaled Identity Subject Specification: (None) | | | | | | |
|  |  |  |  |  |  |  |

Covariance Parameters

Random EffectRandom Effect, table, 2 levels of column headers and 1 levels of row headers, table with 7 columns and 5 rows

|  |  |  |  |  |  |  |
| --- | --- | --- | --- | --- | --- | --- |
| Random Effect Covariance | Estimate | Std. Error | Z | Sig. | 95% Confidence Interval | |
|  |  |  |  |  | Lower | Upper |
| Var(Intercept) | .437 | .269 | 1.625 | .104 | .131 | 1.461 |
|  |  |  |  |  |  |  |
| --- | --- | --- | --- | --- | --- | --- |
| Covariance Structure: Variance components Subject Specification: lekol1 | | | | | | |
|  |  |  |  |  |  |  |

Estimated Marginal Means for Top Significant Fixed Effects

Log  
Log - Log - November 29, 2020

GENLINMIXED  
  /DATA\_STRUCTURE SUBJECTS=lekol1  
  /FIELDS TARGET=anemia TRIALS=NONE  ANALYSIS\_WEIGHT=scale\_weight OFFSET=NONE  
  /TARGET\_OPTIONS REFERENCE=0 DISTRIBUTION=BINOMIAL LINK=LOGIT  
  /FIXED  EFFECTS= age recode\_incnew urbanru recodeworm adultsliving recodestunted adultsliving\*urbanru  
  USE\_INTERCEPT=TRUE  
  /RANDOM USE\_INTERCEPT=TRUE SUBJECTS=lekol1 COVARIANCE\_TYPE=VARIANCE\_COMPONENTS  
  /BUILD\_OPTIONS TARGET\_CATEGORY\_ORDER=ASCENDING INPUTS\_CATEGORY\_ORDER=ASCENDING MAX\_ITERATIONS=100  
  CONFIDENCE\_LEVEL=95 DF\_METHOD=RESIDUAL COVB=MODEL PCONVERGE=0.000001(ABSOLUTE) SCORING=0 SINGULAR=0.000000000001.

Generalized Linear Mixed Models

WarningsWarnings, table, 0 levels of column headers and 0 levels of row headers, table with 1 columns and 4 rows

|  |
| --- |
| glmm: One or more records are not used in the analysis because they have one or more fields with invalid or missing values. |
| glmm: Valid values for events (target) and trials variables are non-negative and positive integers respectively, and the number of trials cannot be less than the number of events. |
| glmm: Analysis weight data values must be positive. |
|  |

Generalized Linear Mixed Models

Case Processing SummaryCase Processing Summary, table, 1 levels of column headers and 1 levels of row headers, table with 3 columns and 5 rows

|  |  |  |
| --- | --- | --- |
|  | N | Percent |
| Included | 1150 | 89.8% |
| Excluded | 131 | 10.2% |
| Total | 1281 | 100.0% |
|  |  |  |

Generalized Linear Mixed Models

Model SummaryModel Summary, table, 0 levels of column headers and 2 levels of row headers, table with 3 columns and 7 rows

|  |  |  |
| --- | --- | --- |
| Target | | Anemia: <11 <5 yrs; <11.5 11.5 yrs; <12 14 yrs |
| Probability Distribution | | Binomial |
| Link Function | | Logit |
| Information Criterion | Akaike Corrected | 5381.946 |
|  | Bayesian | 5386.983 |
|  |  |  |
| --- | --- | --- |
| Information criteria are based on the -2 log likelihood (5379.942) and are used to compare models. Models with smaller information criterion values fit better. | | |
|  |  |  |

Generalized Linear Mixed Models

Data StructureaData Structure, table, 2 levels of column headers and 1 levels of row headers, table with 3 columns and 16 rows

|  |  |  |
| --- | --- | --- |
|  | Subjects | Target |
|  | lekol1 | Anemia: <11 <5 yrs; <11.5 11.5 yrs; <12 14 yrs |
| Data for First Subject | 1 | Non-anemic |
|  | 1 | Anemic |
|  | 1 | Non-anemic |
|  | 1 | Anemic |
|  | 1 | Anemic |
|  | 1 | Non-anemic |
|  | 1 | Non-anemic |
|  | 1 | Non-anemic |
|  | 1 | Non-anemic |
|  | 1 | Anemic |
| Total Number of Levels | 9 |  |
|  |  |  |
| --- | --- | --- |
| Only the first 10 records are displayed.a | | |
| a. Target: Anemia: <11 <5 yrs; <11.5 11.5 yrs; <12 14 yrs | | |
|  |  |  |

Generalized Linear Mixed Models

Generalized Linear Mixed Models

Fixed EffectsaFixed Effects, table, 1 levels of column headers and 1 levels of row headers, table with 5 columns and 12 rows

|  |  |  |  |  |
| --- | --- | --- | --- | --- |
| Source | F | df1 | df2 | Sig. |
| Corrected Model | 7.490 | 7 | 1142 | .000 |
| age | 20.077 | 1 | 1142 | .000 |
| recode\_incnew | 3.780 | 1 | 1142 | .052 |
| urbanru | .384 | 1 | 1142 | .536 |
| recodeworm | 2.987 | 1 | 1142 | .084 |
| adultsliving | 5.381 | 1 | 1142 | .021 |
| recodestunted | 10.292 | 1 | 1142 | .001 |
| adultsliving \* urbanru | 14.692 | 1 | 1142 | .000 |
|  |  |  |  |  |
| --- | --- | --- | --- | --- |
| Probability distribution: Binomial Link function: Logita | | | | |
| a. Target: Anemia: <11 <5 yrs; <11.5 11.5 yrs; <12 14 yrs | | | | |
|  |  |  |  |  |

Generalized Linear Mixed Models

Generalized Linear Mixed Models

Fixed CoefficientsaFixed Coefficients, table, 2 levels of column headers and 1 levels of row headers, table with 10 columns and 19 rows

|  |  |  |  |  |  |  |  |  |  |
| --- | --- | --- | --- | --- | --- | --- | --- | --- | --- |
| Model Term | Coefficient | Std. Error | t | Sig. | 95% Confidence Interval | | Exp(Coefficient) | 95% Confidence Interval for Exp(Coefficient) | |
|  |  |  |  |  | Lower | Upper |  | Lower | Upper |
| Intercept | 1.847 | .4071 | 4.537 | .000 | 1.048 | 2.646 | 6.340 | 2.853 | 14.093 |
| age | -.117 | .0262 | -4.481 | .000 | -.169 | -.066 | .889 | .845 | .936 |
| recode\_incnew=0 | -.269 | .1383 | -1.944 | .052 | -.540 | .002 | .764 | .583 | 1.002 |
| recode\_incnew=1 | 0b | . | . | . | . | . | . | . | . |
| urbanru=0 | .349 | .5627 | .619 | .536 | -.755 | 1.453 | 1.417 | .470 | 4.274 |
| urbanru=1 | 0b | . | . | . | . | . | . | . | . |
| recodeworm=0 | .433 | .2508 | 1.728 | .084 | -.059 | .925 | 1.542 | .943 | 2.523 |
| recodeworm=1 | 0b | . | . | . | . | . | . | . | . |
| adultsliving | .066 | .0553 | 1.194 | .233 | -.042 | .174 | 1.068 | .958 | 1.191 |
| recodestunted=0 | .719 | .2240 | 3.208 | .001 | .279 | 1.158 | 2.052 | 1.322 | 3.184 |
| recodestunted=1 | 0b | . | . | . | . | . | . | . | . |
| adultsliving\*[urbanru=0] | -.334 | .0871 | -3.833 | .000 | -.505 | -.163 | .716 | .604 | .850 |
| adultsliving\*[urbanru=1] | 0b | . | . | . | . | . | . | . | . |
|  |  |  |  |  |  |  |  |  |  |
| --- | --- | --- | --- | --- | --- | --- | --- | --- | --- |
| Probability distribution: Binomial Link function: Logita | | | | | | | | | |
| a. Target: Anemia: <11 <5 yrs; <11.5 11.5 yrs; <12 14 yrs | | | | | | | | | |
| b. This coefficient is set to zero because it is redundant. | | | | | | | | | |
|  |  |  |  |  |  |  |  |  |  |

Generalized Linear Mixed Models

Random Effect Covariances  
Random Effect Covariances - Random Effect Block 1 - November 29, 2020

Random Effect Block 1Random Effect Block 1, table, 1 levels of column headers and 1 levels of row headers, table with 2 columns and 4 rows

|  |  |
| --- | --- |
| Random Effect Block | Intercept |
| Intercept | .437 |
|  |  |
| --- | --- |
| Covariance Structure: Variance components Subject Specification: lekol1 | |
|  |  |

Covariance Parameters

Covariance Parameters SummaryCovariance Parameters Summary, table, 0 levels of column headers and 2 levels of row headers, table with 3 columns and 8 rows

|  |  |  |
| --- | --- | --- |
| Covariance Parameters | Residual Effect | 0 |
|  | Random Effects | 1 |
| Design Matrix Columns | Fixed Effects | 13 |
|  | Random Effects | 1a |
| Common Subjects | | 9 |
|  |  |  |
| --- | --- | --- |
| Common subjects are based on the subject specifications for the residual and random effects and are used to chunk the data for better performance. | | |
| a. This is the number of columns per common subject. | | |
|  |  |  |

Covariance Parameters  
Covariance Parameters - Residual Effect - November 29, 2020

Residual EffectResidual Effect, table, 2 levels of column headers and 1 levels of row headers, table with 7 columns and 5 rows

|  |  |  |  |  |  |  |
| --- | --- | --- | --- | --- | --- | --- |
| Residual Effect | Estimate | Std. Error | Z | Sig. | 95% Confidence Interval | |
|  |  |  |  |  | Lower | Upper |
| Variance | 1.000 | . | . | . | . | . |
|  |  |  |  |  |  |  |
| --- | --- | --- | --- | --- | --- | --- |
| Covariance Structure: Scaled Identity Subject Specification: (None) | | | | | | |
|  |  |  |  |  |  |  |

Covariance Parameters

Random EffectRandom Effect, table, 2 levels of column headers and 1 levels of row headers, table with 7 columns and 5 rows

|  |  |  |  |  |  |  |
| --- | --- | --- | --- | --- | --- | --- |
| Random Effect Covariance | Estimate | Std. Error | Z | Sig. | 95% Confidence Interval | |
|  |  |  |  |  | Lower | Upper |
| Var(Intercept) | .437 | .269 | 1.625 | .104 | .131 | 1.461 |
|  |  |  |  |  |  |  |
| --- | --- | --- | --- | --- | --- | --- |
| Covariance Structure: Variance components Subject Specification: lekol1 | | | | | | |
|  |  |  |  |  |  |  |

Estimated Marginal Means for Top Significant Fixed Effects

Log  
Log - Log - November 29, 2020

GENLINMIXED  
  /DATA\_STRUCTURE SUBJECTS=lekol1  
  /FIELDS TARGET=anemia TRIALS=NONE  ANALYSIS\_WEIGHT=scale\_weight OFFSET=NONE  
  /TARGET\_OPTIONS REFERENCE=0 DISTRIBUTION=BINOMIAL LINK=LOGIT  
  /FIXED  EFFECTS= age recode\_incnew rural\_urban recodeworm adultsliving recodestunted adultsliving\*rural\_urban recodeworm\*rural\_urban recodestunted\*rural\_urban  
  USE\_INTERCEPT=TRUE  
  /RANDOM USE\_INTERCEPT=TRUE SUBJECTS=lekol1 COVARIANCE\_TYPE=VARIANCE\_COMPONENTS  
  /BUILD\_OPTIONS TARGET\_CATEGORY\_ORDER=ASCENDING INPUTS\_CATEGORY\_ORDER=ASCENDING MAX\_ITERATIONS=100  
  CONFIDENCE\_LEVEL=95 DF\_METHOD=RESIDUAL COVB=MODEL PCONVERGE=0.000001(ABSOLUTE) SCORING=0 SINGULAR=0.000000000001.

Generalized Linear Mixed Models

WarningsWarnings, table, 0 levels of column headers and 0 levels of row headers, table with 1 columns and 4 rows

|  |
| --- |
| glmm: One or more records are not used in the analysis because they have one or more fields with invalid or missing values. |
| glmm: Valid values for events (target) and trials variables are non-negative and positive integers respectively, and the number of trials cannot be less than the number of events. |
| glmm: Analysis weight data values must be positive. |
|  |

Generalized Linear Mixed Models

Case Processing SummaryCase Processing Summary, table, 1 levels of column headers and 1 levels of row headers, table with 3 columns and 5 rows

|  |  |  |
| --- | --- | --- |
|  | N | Percent |
| Included | 1150 | 89.8% |
| Excluded | 131 | 10.2% |
| Total | 1281 | 100.0% |
|  |  |  |

Generalized Linear Mixed Models

Model SummaryModel Summary, table, 0 levels of column headers and 2 levels of row headers, table with 3 columns and 7 rows

|  |  |  |
| --- | --- | --- |
| Target | | Anemia: <11 <5 yrs; <11.5 11.5 yrs; <12 14 yrs |
| Probability Distribution | | Binomial |
| Link Function | | Logit |
| Information Criterion | Akaike Corrected | 5374.265 |
|  | Bayesian | 5379.301 |
|  |  |  |
| --- | --- | --- |
| Information criteria are based on the -2 log likelihood (5372.262) and are used to compare models. Models with smaller information criterion values fit better. | | |
|  |  |  |

Generalized Linear Mixed Models

Data StructureaData Structure, table, 2 levels of column headers and 1 levels of row headers, table with 3 columns and 16 rows

|  |  |  |
| --- | --- | --- |
|  | Subjects | Target |
|  | lekol1 | Anemia: <11 <5 yrs; <11.5 11.5 yrs; <12 14 yrs |
| Data for First Subject | 1 | Non-anemic |
|  | 1 | Anemic |
|  | 1 | Non-anemic |
|  | 1 | Anemic |
|  | 1 | Anemic |
|  | 1 | Non-anemic |
|  | 1 | Non-anemic |
|  | 1 | Non-anemic |
|  | 1 | Non-anemic |
|  | 1 | Anemic |
| Total Number of Levels | 9 |  |
|  |  |  |
| --- | --- | --- |
| Only the first 10 records are displayed.a | | |
| a. Target: Anemia: <11 <5 yrs; <11.5 11.5 yrs; <12 14 yrs | | |
|  |  |  |

Generalized Linear Mixed Models

Generalized Linear Mixed Models

Fixed EffectsaFixed Effects, table, 1 levels of column headers and 1 levels of row headers, table with 5 columns and 14 rows

|  |  |  |  |  |
| --- | --- | --- | --- | --- |
| Source | F | df1 | df2 | Sig. |
| Corrected Model | 6.176 | 9 | 1140 | .000 |
| age | 20.233 | 1 | 1140 | .000 |
| recode\_incnew | 3.679 | 1 | 1140 | .055 |
| rural\_urban | .000 | 1 | 1140 | .995 |
| recodeworm | .053 | 1 | 1140 | .819 |
| adultsliving | 5.360 | 1 | 1140 | .021 |
| recodestunted | 10.138 | 1 | 1140 | .001 |
| adultsliving \* rural\_urban | 14.986 | 1 | 1140 | .000 |
| recodeworm \* rural\_urban | 1.782 | 1 | 1140 | .182 |
| recodestunted \* rural\_urban | 2.432 | 1 | 1140 | .119 |
|  |  |  |  |  |
| --- | --- | --- | --- | --- |
| Probability distribution: Binomial Link function: Logita | | | | |
| a. Target: Anemia: <11 <5 yrs; <11.5 11.5 yrs; <12 14 yrs | | | | |
|  |  |  |  |  |

Generalized Linear Mixed Models

Generalized Linear Mixed Models  
Including all intraction terms Generalized Linear Mixed Models - Fixed Coefficients - November 29, 2(more)020(less)

Fixed CoefficientsaFixed Coefficients, table, 2 levels of column headers and 1 levels of row headers, table with 10 columns and 27 rows

|  |  |  |  |  |  |  |  |  |  |
| --- | --- | --- | --- | --- | --- | --- | --- | --- | --- |
| Model Term | Coefficient | Std. Error | t | Sig. | 95% Confidence Interval | | Exp(Coefficient) | 95% Confidence Interval for Exp(Coefficient) | |
|  |  |  |  |  | Lower | Upper |  | Lower | Upper |
| Intercept | 2.191 | .5088 | 4.306 | .000 | 1.193 | 3.189 | 8.945 | 3.296 | 24.275 |
| age | -.118 | .0263 | -4.498 | .000 | -.170 | -.067 | .888 | .844 | .935 |
| recode\_incnew=0 | -.266 | .1387 | -1.918 | .055 | -.538 | .006 | .766 | .584 | 1.006 |
| recode\_incnew=1 | 0b | . | . | . | . | . | . | . | . |
| rural\_urban=0 | -.320 | .5657 | -.566 | .572 | -1.430 | .790 | .726 | .239 | 2.203 |
| rural\_urban=1 | 0b | . | . | . | . | . | . | . | . |
| recodeworm=0 | -.770 | .9483 | -.812 | .417 | -2.631 | 1.090 | .463 | .072 | 2.975 |
| recodeworm=1 | 0b | . | . | . | . | . | . | . | . |
| adultsliving | -.271 | .0678 | -3.997 | .000 | -.404 | -.138 | .763 | .668 | .871 |
| recodestunted=0 | 1.045 | .3171 | 3.295 | .001 | .423 | 1.667 | 2.843 | 1.526 | 5.295 |
| recodestunted=1 | 0b | . | . | . | . | . | . | . | . |
| adultsliving\*[rural\_urban=0] | .339 | .0877 | 3.871 | .000 | .167 | .511 | 1.404 | 1.182 | 1.667 |
| adultsliving\*[rural\_urban=1] | 0b | . | . | . | . | . | . | . | . |
| [recodeworm=0]\*[rural\_urban=0] | 1.315 | .9850 | 1.335 | .182 | -.618 | 3.247 | 3.724 | .539 | 25.722 |
| [recodeworm=1]\*[rural\_urban=0] | 0b | . | . | . | . | . | . | . | . |
| [recodeworm=0]\*[rural\_urban=1] | 0b | . | . | . | . | . | . | . | . |
| [recodeworm=1]\*[rural\_urban=1] | 0b | . | . | . | . | . | . | . | . |
| [recodestunted=0]\*[rural\_urban=0] | -.685 | .4390 | -1.560 | .119 | -1.546 | .177 | .504 | .213 | 1.193 |
| [recodestunted=1]\*[rural\_urban=0] | 0b | . | . | . | . | . | . | . | . |
| [recodestunted=0]\*[rural\_urban=1] | 0b | . | . | . | . | . | . | . | . |
| [recodestunted=1]\*[rural\_urban=1] | 0b | . | . | . | . | . | . | . | . |
|  |  |  |  |  |  |  |  |  |  |
| --- | --- | --- | --- | --- | --- | --- | --- | --- | --- |
| Probability distribution: Binomial Link function: Logita | | | | | | | | | |
| a. Target: Anemia: <11 <5 yrs; <11.5 11.5 yrs; <12 14 yrs | | | | | | | | | |
| b. This coefficient is set to zero because it is redundant. | | | | | | | | | |
|  |  |  |  |  |  |  |  |  |  |

Generalized Linear Mixed Models

Random Effect Covariances  
Random Effect Covariances - Random Effect Block 1 - November 29, 2020

Random Effect Block 1Random Effect Block 1, table, 1 levels of column headers and 1 levels of row headers, table with 2 columns and 4 rows

|  |  |
| --- | --- |
| Random Effect Block | Intercept |
| Intercept | .438 |
|  |  |
| --- | --- |
| Covariance Structure: Variance components Subject Specification: lekol1 | |
|  |  |

Covariance Parameters

Covariance Parameters SummaryCovariance Parameters Summary, table, 0 levels of column headers and 2 levels of row headers, table with 3 columns and 8 rows

|  |  |  |
| --- | --- | --- |
| Covariance Parameters | Residual Effect | 0 |
|  | Random Effects | 1 |
| Design Matrix Columns | Fixed Effects | 21 |
|  | Random Effects | 1a |
| Common Subjects | | 9 |
|  |  |  |
| --- | --- | --- |
| Common subjects are based on the subject specifications for the residual and random effects and are used to chunk the data for better performance. | | |
| a. This is the number of columns per common subject. | | |
|  |  |  |

Covariance Parameters  
Covariance Parameters - Residual Effect - November 29, 2020

Residual EffectResidual Effect, table, 2 levels of column headers and 1 levels of row headers, table with 7 columns and 5 rows

|  |  |  |  |  |  |  |
| --- | --- | --- | --- | --- | --- | --- |
| Residual Effect | Estimate | Std. Error | Z | Sig. | 95% Confidence Interval | |
|  |  |  |  |  | Lower | Upper |
| Variance | 1.000 | . | . | . | . | . |
|  |  |  |  |  |  |  |
| --- | --- | --- | --- | --- | --- | --- |
| Covariance Structure: Scaled Identity Subject Specification: (None) | | | | | | |
|  |  |  |  |  |  |  |

Covariance Parameters

Random EffectRandom Effect, table, 2 levels of column headers and 1 levels of row headers, table with 7 columns and 5 rows

|  |  |  |  |  |  |  |
| --- | --- | --- | --- | --- | --- | --- |
| Random Effect Covariance | Estimate | Std. Error | Z | Sig. | 95% Confidence Interval | |
|  |  |  |  |  | Lower | Upper |
| Var(Intercept) | .438 | .269 | 1.624 | .104 | .131 | 1.463 |
|  |  |  |  |  |  |  |
| --- | --- | --- | --- | --- | --- | --- |
| Covariance Structure: Variance components Subject Specification: lekol1 | | | | | | |
|  |  |  |  |  |  |  |

Estimated Marginal Means for Top Significant Fixed Effects

Log  
Log - Log - November 29, 2020

USE ALL.  
COMPUTE filter\_$=(rural\_urban = 1).  
VARIABLE LABELS filter\_$ 'rural\_urban = 1 (FILTER)'.  
VALUE LABELS filter\_$ 0 'Not Selected' 1 'Selected'.  
FORMATS filter\_$ (f1.0).  
FILTER BY filter\_$.  
EXECUTE.  
FREQUENCIES VARIABLES=travay\_r  
  /STATISTICS=RANGE MINIMUM MAXIMUM MODE  
  /ORDER=ANALYSIS.

Frequencies

StatisticsStatistics, table, Respondent Occupation, 1 layers, 0 levels of column headers and 2 levels of row headers, table with 3 columns and 8 rows

| |  |  | | --- | --- | | Respondent Occupation | Respondent Occupation | | | |
|  |  |  |
| --- | --- | --- |
| N | Valid | 293 |
|  | Missing | 7 |
| Mode | | 7 |
| Range | | 11 |
| Minimum | | 1 |
| Maximum | | 12 |
|  |  |  |

Frequencies

Respondent OccupationRespondent Occupation, table, 1 levels of column headers and 2 levels of row headers, table with 6 columns and 10 rows

|  |  |  |  |  |  |
| --- | --- | --- | --- | --- | --- |
|  | | Frequency | Percent | Valid Percent | Cumulative Percent |
| Valid | Market | 95 | 31.7 | 32.4 | 32.4 |
|  | farming/ cultivation | 104 | 34.7 | 35.5 | 67.9 |
|  | teacher | 9 | 3.0 | 3.1 | 71.0 |
|  | unemployed | 69 | 23.0 | 23.5 | 94.5 |
|  | other | 16 | 5.3 | 5.5 | 100.0 |
|  | Total | 293 | 97.7 | 100.0 |  |
| Missing | System | 7 | 2.3 |  |  |
| Total | | 300 | 100.0 |  |  |
|  |  |  |  |  |  |

Log

FREQUENCIES VARIABLES=travay\_m  
  /STATISTICS=RANGE MINIMUM MAXIMUM MODE  
  /ORDER=ANALYSIS.

Frequencies

StatisticsStatistics, table, Spouse Occupation, 1 layers, 0 levels of column headers and 2 levels of row headers, table with 3 columns and 8 rows

| |  |  | | --- | --- | | Spouse Occupation | Spouse Occupation | | | |
|  |  |  |
| --- | --- | --- |
| N | Valid | 297 |
|  | Missing | 3 |
| Mode | | 7 |
| Range | | 11 |
| Minimum | | 1 |
| Maximum | | 12 |
|  |  |  |

Frequencies

Spouse OccupationSpouse Occupation, table, 1 levels of column headers and 2 levels of row headers, table with 6 columns and 12 rows

|  |  |  |  |  |  |
| --- | --- | --- | --- | --- | --- |
|  | | Frequency | Percent | Valid Percent | Cumulative Percent |
| Valid | Market | 21 | 7.0 | 7.1 | 7.1 |
|  | factory | 6 | 2.0 | 2.0 | 9.1 |
|  | farming/ cultivation | 157 | 52.3 | 52.9 | 62.0 |
|  | teacher | 13 | 4.3 | 4.4 | 66.3 |
|  | unemployed | 23 | 7.7 | 7.7 | 74.1 |
|  | construction | 34 | 11.3 | 11.4 | 85.5 |
|  | other | 43 | 14.3 | 14.5 | 100.0 |
|  | Total | 297 | 99.0 | 100.0 |  |
| Missing | System | 3 | 1.0 |  |  |
| Total | | 300 | 100.0 |  |  |
|  |  |  |  |  |  |

Log

USE ALL.  
COMPUTE filter\_$=(rural\_urban = 0).  
VARIABLE LABELS filter\_$ 'rural\_urban = 0 (FILTER)'.  
VALUE LABELS filter\_$ 0 'Not Selected' 1 'Selected'.  
FORMATS filter\_$ (f1.0).  
FILTER BY filter\_$.  
EXECUTE.  
FREQUENCIES VARIABLES=travay\_r  
  /STATISTICS=RANGE MINIMUM MAXIMUM MODE  
  /ORDER=ANALYSIS.

Frequencies

StatisticsStatistics, table, Respondent Occupation, 1 layers, 0 levels of column headers and 2 levels of row headers, table with 3 columns and 8 rows

| |  |  | | --- | --- | | Respondent Occupation | Respondent Occupation | | | |
|  |  |  |
| --- | --- | --- |
| N | Valid | 882 |
|  | Missing | 99 |
| Mode | | 1 |
| Range | | 11 |
| Minimum | | 1 |
| Maximum | | 12 |
|  |  |  |

Frequencies

Respondent OccupationRespondent Occupation, table, 1 levels of column headers and 2 levels of row headers, table with 6 columns and 16 rows

|  |  |  |  |  |  |
| --- | --- | --- | --- | --- | --- |
|  | | Frequency | Percent | Valid Percent | Cumulative Percent |
| Valid | Market | 467 | 47.6 | 52.9 | 52.9 |
|  | Business (shop) | 8 | .8 | .9 | 53.9 |
|  | Office admin | 5 | .5 | .6 | 54.4 |
|  | fishing | 2 | .2 | .2 | 54.6 |
|  | livestock | 2 | .2 | .2 | 54.9 |
|  | farming/ cultivation | 11 | 1.1 | 1.2 | 56.1 |
|  | teacher | 18 | 1.8 | 2.0 | 58.2 |
|  | maid/servant/janitor | 32 | 3.3 | 3.6 | 61.8 |
|  | unemployed | 169 | 17.2 | 19.2 | 81.0 |
|  | construction | 46 | 4.7 | 5.2 | 86.2 |
|  | other | 122 | 12.4 | 13.8 | 100.0 |
|  | Total | 882 | 89.9 | 100.0 |  |
| Missing | System | 99 | 10.1 |  |  |
| Total | | 981 | 100.0 |  |  |
|  |  |  |  |  |  |

Log

FREQUENCIES VARIABLES=travay\_m  
  /STATISTICS=RANGE MINIMUM MAXIMUM MODE  
  /ORDER=ANALYSIS.

Frequencies

StatisticsStatistics, table, Spouse Occupation, 1 layers, 0 levels of column headers and 2 levels of row headers, table with 3 columns and 8 rows

| |  |  | | --- | --- | | Spouse Occupation | Spouse Occupation | | | |
|  |  |  |
| --- | --- | --- |
| N | Valid | 880 |
|  | Missing | 101 |
| Mode | | 12 |
| Range | | 11 |
| Minimum | | 1 |
| Maximum | | 12 |
|  |  |  |

Frequencies

Spouse OccupationSpouse Occupation, table, 1 levels of column headers and 2 levels of row headers, table with 6 columns and 17 rows

|  |  |  |  |  |  |
| --- | --- | --- | --- | --- | --- |
|  | | Frequency | Percent | Valid Percent | Cumulative Percent |
| Valid | Market | 193 | 19.7 | 21.9 | 21.9 |
|  | Business (shop) | 35 | 3.6 | 4.0 | 25.9 |
|  | Office admin | 11 | 1.1 | 1.3 | 27.2 |
|  | factory | 7 | .7 | .8 | 28.0 |
|  | fishing | 5 | .5 | .6 | 28.5 |
|  | livestock | 2 | .2 | .2 | 28.8 |
|  | farming/ cultivation | 70 | 7.1 | 8.0 | 36.7 |
|  | teacher | 23 | 2.3 | 2.6 | 39.3 |
|  | maid/servant/janitor | 10 | 1.0 | 1.1 | 40.5 |
|  | unemployed | 97 | 9.9 | 11.0 | 51.5 |
|  | construction | 144 | 14.7 | 16.4 | 67.8 |
|  | other | 283 | 28.8 | 32.2 | 100.0 |
|  | Total | 880 | 89.7 | 100.0 |  |
| Missing | System | 101 | 10.3 |  |  |
| Total | | 981 | 100.0 |  |  |
|  |  |  |  |  |  |

IBM SPSS Web Report

X

ABOUT

|  |
| --- |
| Created Using: IBM SPSS Statistics 26 |
| Creation Date: Nov 29, 2020 |
| Document Version: OriginalSaved Copy |
| Saved Date:  Nov 29, 2020 |

Navigation Controls

|  |
| --- |
| Contents - Opens and closes the list of charts and tables in the Web Report |
| Next & Previous - Display the next or previous table or chart in the Web Report |
| Help - Opens Help |

Toolbar Buttons

|  |  |
| --- | --- |
|  | Undo - Undoes the last change in the document. |
|  | Edit - Open the Editor tool for tables and charts. Certain editing options are only available when you are connected to an Internet server. |
|  | Save - Creates a new copy of the Web Report with the saved changes. |
|  | Print - Prints the current object when in Object View and all objects in Page View. |
|  | Page View - Switches the Web Report to display all the tables and charts on a single page. |
|  | Object View - Switches the Web Report so that each table or chart is displayed one at a time. |

Connecting to a Server

:   The status of the Web Report's connection to an Internet server appears in the top right corner of the Web Report.
:   An Internet connection is not required to open a Web Report. With a saved copy of the Web Report you can view all of the charts and tables, and have some limited editing ability, when not connected to the Internet.
:   Connecting a Web Report to an Internet server will enable far greater editing capabilities for tables and for charts.

- If the author specified an Internet server when they created the Web Report, the Web Report will attempt to connect to the server automatically when it is opened.
- If the Web Report does not connect to a server, click on the server Status Message to open tools to retry the connection, try a different server, or enter a new server address.
- For information about adding the enhanced controls to your Internet Server, go to https://developer.ibm.com/predictiveanalytics.
- If you specify a new server connection, the preferred format is http://xxx.xxx.xxx.xxx:xxxx.

Editing Tables

|  |  |
| --- | --- |
| Some of this functionality is only available when connected to an Internet server. | |
|  | Create a chart - Create a chart from the selected cells in the table. |
|  | Pivot and Sort - Transpose, sort, and pivot the table. |
|  | Background color - The background color of the selected cells. |
|  | Text Color and Style - Font color, style, and size. |
|  | Number Format - Font color, style, and size. |

Editing Charts

|  |  |
| --- | --- |
| All of this functionality is only available when connected to an Internet server. | |
|  | Chart Size - Change the height and width of the chart |
|  | Background color - The background color of the selected object. |
|  | Border and Line Style - The color and thickness of the line or border. |
|  | Text Color and Style - Font color, style, and size. |
|  | Number Format - Font color, style, and size. |
|  | Axis Properties - Change the scale and display axis titles and ticks. |

Add a chart

Pivot and Sort

Chart Size   
  

|  |  |  |
| --- | --- | --- |
|  |  |  |
|  |  |  |
| Lock aspect ratio | | |

Background   

|  |  |  |  |  |  |
| --- | --- | --- | --- | --- | --- |
|  | |  | |  | |
|  |  |  |  |  |  |
|  |  |  |  |  |  |
|  |  |  |  |  |  |

Line and Borders   

|  |  |  |  |  |  |
| --- | --- | --- | --- | --- | --- |
|  | |  | |  | |
|  |  |  |  |  |  |
|  |  |  |  |  |  |
|  |  |  |  |  |  |

Text Format   

|  |  |  |  |  |  |
| --- | --- | --- | --- | --- | --- |
|  | |  | |  | |
|  |  |  |  |  |  |
|  |  |  |  |  |  |
|  |  |  |  |  |  |

  

|  |  |  |
| --- | --- | --- |
|  |  |  |

  

|  |  |  |  |
| --- | --- | --- | --- |
|  |  |  | Font Family  Adobe Devanagari Arial Arial Black Arial Narrow Bahnschrift Book Antiqua Bookman Old Style Bookshelf Symbol 7 Bradley Hand ITC Calibri Calibri Light Cambria Cambria Math Candara Candara Light Century Century Gothic Comic Sans MS Consolas Constantia Corbel Corbel Light Courier New Dialog DialogInput Ebrima Franklin Gothic Medium Freestyle Script French Script MT Frutiger LT Pro 45 Light Gabriola Gadugi Garamond Georgia HelvNeue Roman for IBM HoloLens MDL2 Assets Impact Ink Free Javanese Text Juice ITC Kristen ITC Leelawadee UI Leelawadee UI Semilight Lucida Bright Lucida Console Lucida Handwriting Lucida Sans Lucida Sans Typewriter Lucida Sans Unicode Malgun Gothic Malgun Gothic Semilight Marlett Microsoft Himalaya Microsoft JhengHei Microsoft JhengHei Light Microsoft JhengHei UI Microsoft JhengHei UI Light Microsoft New Tai Lue Microsoft PhagsPa Microsoft Sans Serif Microsoft Tai Le Microsoft YaHei Microsoft YaHei Light Microsoft YaHei UI Microsoft YaHei UI Light Microsoft Yi Baiti MingLiU-ExtB MingLiU\_HKSCS-ExtB Mistral Mongolian Baiti Monospaced Monotype Corsiva MS Gothic MS PGothic MS Reference Sans Serif MS Reference Specialty MS UI Gothic MT Extra MV Boli Myanmar Text Nirmala UI Nirmala UI Semilight NSimSun Palatino Linotype Papyrus PMingLiU-ExtB Pristina SansSerif Segoe MDL2 Assets Segoe Print Segoe Script Segoe UI Segoe UI Black Segoe UI Emoji Segoe UI Historic Segoe UI Light Segoe UI Semibold Segoe UI Semilight Segoe UI Symbol Serif SimSun SimSun-ExtB Sitka Banner Sitka Display Sitka Heading Sitka Small Sitka Subheading Sitka Text Sylfaen Symbol Tahoma Tempus Sans ITC Times New Roman Trebuchet MS Verdana Webdings Wingdings Wingdings 2 Wingdings 3 Yu Gothic Yu Gothic Light Yu Gothic Medium Yu Gothic UI Yu Gothic UI Light Yu Gothic UI Semibold Yu Gothic UI Semilight ZWAdobeF |

Number Format   
  

|  |  |  |
| --- | --- | --- |
| 0.00 |  |  |

Axis Options   
  

|  |  |  |
| --- | --- | --- |
|  |  |  |
|  |  |  |
| Display Axis Title | | | |
| Display Ticks | | | |
