# Supplementary material for: Differences in factors associated with anemia in Haitian children from urban and rural areas
Source: PLoS One. 2021 Apr 6;16(4):e0247975. doi: 10.1371/journal.pone.0247975 (PMC8023464; doi:10.1371/journal.pone.0247975)
Supplement: S2 File — (DOCX) [file pone.0247975.s003.docx]

Study Profile:

Total n=981

Incomplete baseline survey n=188

Did not meet eligibility criteria n=17

Assessed for eligibility n=1,186

Total n=300

Did not meet age criteria n=142

Assessed for eligibility n=442

URBAN TRIAL

RURAL TRIAL
